# Foam Cell Macrophages in Tuberculosis

**DOI:** 10.3389/fimmu.2021.775326

**Published:** 2021-12-15

**Authors:** Pooja Agarwal, Siamon Gordon, Fernando O. Martinez

**Affiliations:** ^1^ Faculty of Health and Medical Sciences, University of Surrey, Guildford, United Kingdom; ^2^ Graduate Institute of Biomedical Sciences, College of Medicine, Chang Gung University, Taoyuan City, Taiwan; ^3^ Sir William Dunn School of Pathology, University of Oxford, Oxford, United Kingdom

**Keywords:** tuberculosis, *Mycobacterium*, macrophage, foam cells, lipid droplets

## Abstract

*Mycobacterium tuberculosis* infects primarily macrophages in the lungs. Infected macrophages are surrounded by other immune cells in well organised structures called granulomata. As part of the response to TB, a type of macrophage loaded with lipid droplets arises which we call Foam cell macrophages. They are macrophages filled with lipid laden droplets, which are synthesised in response to increased uptake of extracellular lipids, metabolic changes and infection itself. They share the appearance with atherosclerosis foam cells, but their lipid contents and roles are different. In fact, lipid droplets are immune and metabolic organelles with emerging roles in Tuberculosis. Here we discuss lipid droplet and foam cell formation, evidence regarding the inflammatory and immune properties of foam cells in TB, and address gaps in our knowledge to guide further research.

## Introduction

Tuberculosis (TB) is a chronic infectious disease caused by the bacterium *Mycobacterium tuberculosis* (Mtb). TB mortality rates increase with weaker immune systems caused by comorbidities such as diabetes, acquired immune deficiency syndrome (AIDS), smoking, alcohol consumption, and undernutrition (WHO, Global Tuberculosis report 2020). TB is one of the deadliest diseases worldwide, especially in developing countries in communities with low income and poor nutrition. The WHO predicted that COVID will lead to a new TB crisis and anticipates an increase in TB deaths worldwide due to late diagnosis and treatment during peaks of the pandemic.

Macrophages are crucial in the response to Mtb and have the main role of containing and killing the pathogen. The main cytokine associated with Mtb killing and macrophage activation is IFN-γ. Other prototypic inflammatory cytokines such as IL-12, and the inflammasome regulated IL-1β and IL-18 are also associated with macrophage responses to Mtb. Despite activation programmes, Mtb infects and kills many macrophages, and the pathogen survives inside death resistant macrophages, which are not well defined. Mtb utilises several strategies to persist inside macrophages. For example the pathogen inhibits phagolysosome maturation and acidification by stopping translocation of enzymes, inhibition of lysosomal enzymes, and by depleting calcium and hydrogen ions required for the fusion of the lysosome with the phagosome (1).

Mtb infected macrophages orchestrate a pro-inflammatory response leading to granuloma formation. Through TLR and pattern recognition receptor mediated activation (2), Mtb also alters inflammatory pathways and oxidative stress, inducing various forms of cell death and autophagy (1). Thus, cell death and abundant cellular material are hallmarks of the Mtb infection. Foam cells represent a predominant macrophage phenotype observed in TB granulomata surrounding necrotic material.

Foam cell appearance is due to the presence of lipid droplets in the cytoplasm, which grow and are consumed by the cells, as required. Lipid droplets are numerous in macrophages, similar to droplets in brown and beige adipose tissue. TB foam cells share similarities with macrophages that appear in other lipid disorders such as atherosclerosis (also termed foam cells), but their contents in TB are predominantly triglycerides and not cholesterol. The link between this lipid rich profile and the conventional IFN-γ Th1-macrophage response necessary to control Mtb, is ill defined.

Granulomata (plural of granuloma) are organised cellular foci that contain infected macrophages, granulocytes and T lymphocytes, encased by a fibrotic ring. *In vivo*, the various stages of granuloma include nascent, caseous, fibrocaseous and resolved states. Despite such variety, granulomata are always dominated by macrophages. Granulomata with a Th1/M1-like profile have been proposed to control the disease, whereas a Th2/M2 rich profile is associated with exacerbation (3). TB foam cells are predominant features of advanced mycobacterial granulomata associated with caseation. The caseum contains an abundance of triacylglycerols, cholesterol, cholesteryl esters and lactosylceramide, which derive mainly from dead cells (4), becoming the predominant component of foam cells themselves.

Using TB+ human lymph node samples, Peyron et al. showed that foam cells are always close to the necrotic core with a strong correlation, and appear infected by Mtb, as confirmed by Ziehl-Neelson acid-fast bacilli (AFB) positive staining of tissue sections (5). *In vitro*, using PBMC activation assays, they found that Mtb and *Mycobacterium avium, but* not *Mycobacterium smegmatis*, induce foam cell formation, ascribed to the presence of Mycolic acids. However, the mechanism of foam cell formation and the nature of the lipids driving this process are not clear since they did not demonstrate reproducible Mtb replication in foam cells, and in bulk. They also showed bacteria inside large lipid droplets by electron microscopy, but the data need further verification due to the fusion of lipids in processing samples for EM, and the challenge of studying subcellular events with pathogenic bacteria.

We can learn from atherosclerosis and adipose tissue research, since lipid droplet proteins and mechanisms are somewhat conserved. Progression of granulomata to caseation is associated with upregulation of lipid handling proteins such as ADFP, required for droplet formation, Acyl-CoA Synthetase Long-Chain Family Member (ACSL1), involved in the *de novo* triglyceride synthesis pathway and the membranous lysosomal protein Saposin C (SapC), required to remodel the droplets (4). These three proteins involved in lipid metabolism were highly expressed in caseous and fibrocaseous granulomata. SapC was markedly positive in nascent granulomata, whereas ADFP was more prevalent in the foamy macrophage-rich centre of advanced disease, pointing to accumulation of lipid laden foam cells in granuloma formation, over time (4). LTA4H, which synthesises the proinflammatory mediator LTB4, is also highly expressed in the ring of foam cells. The interconversion between macrophages and foam cells and the migration and distribution of macrophages within granulomata deserve further scrutiny.

## Biochemical Pathways Leading to Foam Cell Formation in TB

The main requirement for lipid droplet formation is the increase in neutral lipids in the cell. For uptake of exogenous lipid particles such as lipoproteins and fatty acid complexes, macrophages are equipped with a variety of receptors including scavenger receptors CD36, SR-A1, and SR-B1, the oxidized low-density lipoprotein receptor 1 (LOX-1), Macrophage receptor with collagenous structure (MARCO), the Fatty acid transport protein 1 (FATP1) and Lipoprotein lipase (LPL) (6). The interaction of macrophages with necrotic and apoptotic debris involves a distinct type of immune phagocytic synapse. Intracellular trafficking of fatty acid is supported by another group of proteins including the fatty acid binding proteins, FABP4 and FABP5 (7). CD36 is of particular interest due to its regulation and its role in the recognition of cellular debris, apoptotic cells, and oxidized LDL (oxLDL) (8, 9).

**Figure 1:**
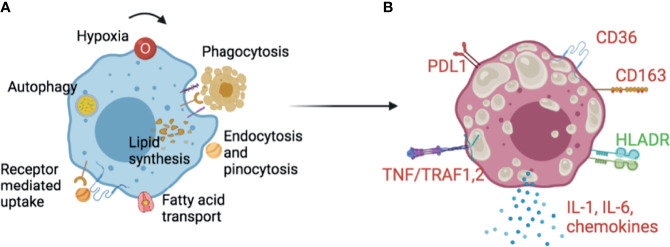
Foam cells in TB. **(A)** Lipid droplet synthesis in macrophages can be triggered by external and internal stimuli. Extrinsic complex sources of lipids include phagocytosis of apoptotic or necrotic cells, or endocytosis, pinocytosis and receptor mediated uptake of lipoproteins. Simpler fatty acids can be transported by specialised machineries in the cell. Lysosomal activity and autophagy contribute to the degradation of accumulated lipid remnants in the cell to avoid lipotoxicity. Cytokines, hormones, growth factors and metabolic changes such as variations of glucose level, induce enzymes and proteins important for lipid synthesis and droplet stability. **(B)** Although there is no consensus, some markers appear repeatedly in Foam cell literature. Foam cells have been shown to have increased scavenger receptors- CD36, CD163, TNF-α/TRAF1,2, the inflammatory cytokine IL-6 and the inflammasome dependent IL-1β. The checkpoint inhibitor PDL1 has been shown as increased while the antigen presentation related HLA-DR as decreased. Upregulated markers are colour coded in red and downregulated in green. These and other markers can guide needed translational histological studies on foam cells in multiple species and cell line models. It is early to ascertain the true nature of foam cells in TB, and more research in primary models is necessary since THP1 and primary macrophages behave differently. See text for references.

Lipid droplets accumulate in the endoplasmic reticulum as the neutral lipid content between leaflets of the ER membrane reaches a high concentration (10). Triglycerides and their components need to be synthesised in order for cells to accumulate lipid droplets in TB. Fatty acids are synthesized *de novo* or more likely, incorporated from the extracellular space, e.g. by lipolysis of lipoproteins and dead cell debris, **Figure 1A**. The process is actively controlled by multiple isoforms of lipid enzymes and accessory proteins reviewed in detail in (11). Internalised complex lipids are degraded to fatty acids by the action of lysosomal acid lipases (LAL) in phagolysosomes (6). Lipophagy degrades particles by various hydrolases into basic components such as amino acids, glucose, nucleotides and free fatty acids (12, 13). Engulfment of lipids from necrotic cells induces triacylglyceride (TAG)- enriched lipid droplets, whereas inhibition of necrosis by the oxidation inhibitor IM54 prevents lipid droplet accumulation. The source of lipid is mainly esterified-lipid of necrotic cells, first degraded by lysosomes then mobilised into TAG, since inhibition of lysosomal lipases by chlorpromazine (CPZ) prevents the transfer of labelled fatty acids of necrotic cells into the TAG of recipient cells (14). **Figure 1B** provides an overall picture of foam cell markers with high scavenger receptors CD36, CD163, TNF receptors and T cell response inhibitors, and decreased HLADR expression.

Triacylglyceride synthesis (TAG) requires *de novo* fatty acid production from cytosolic citrate. This step is produced by the Krebs cycle from oxaloacetate and acetyl-CoA by citrate synthase (CS) and exported from mitochondria through the citrate carrier (CIC). Citrate is broken down by the ATP-citrate synthase-(ACLY), to oxaloacetate and acetyl-CoA which is then used as a substrate for fatty acid and cholesterol synthesis (15). The Fatty Acyl Co-A synthetase (ACS) catalyses the formation of a thioester bond between a fatty acid and coenzyme-A to form fatty Acyl Co-A. Through the successive enzymatic actions of glycerol-3-phosphate acyltransferase (GPAT), 1-acylglycerol-3-phosphate O-acyltransferase (AGPAT) and phosphatidic acid phosphatase (PAP), fatty Acyl Co-A is esterified with Glycerol-3-phosphate to form Diacylglycerol (DAG). In the final step, DAG is esterified to TAG by the Acyl-CoA: diacylglycerol acyltransferases-(DGAT1) and (DGAT2). DGAT1 is exclusively in the ER as a dimer or tetramer form, whereas DGAT2, in addition to the ER, localizes in droplets directly, as well as in mitochondria. DGAT1 preferentially uses exogenous FA while DGAT2 uses fatty acids of both exogenous and endogenous origin (16).

Specific to Mtb infection and inspired by the strong association between foam cells and necrotic areas, Jaisinghani et al. investigated the role of necrotic mixtures and their impact on inflammation, over all. In guinea pigs, they found foam cells close to necrotic areas and enrichment of the expression of TNF-α, *in situ*. *In vitro*, overexpression of DGAT1 in human THP1 cells lead to lipid droplet formation. Knockdown of DGAT1 by shRNA, lead to a decrease in TAG level with a concomitant decrease in quantity and size of lipid droplets in THP1 cells (14). Furthermore, inhibition of DGAT1 by its inhibitor T863 established in (17), precluded lipid droplet formation in IFN-γ activated and Mtb infected bone marrow derived mouse (BMDM) and human macrophages (18). Surprisingly, the key step controlled by fatty acid synthase (FASN), was downregulated in IFN-γ activated and Mtb infected macrophages. However, this did not affect droplet formation, which suggests that TAG synthesis and not the *de novo* synthesis of fatty acid is a major contributor to lipid droplets in Mtb infected macrophages (18).

Acetyl-CoA levels are also regulated by the rate-limiting enzymes Acetyl-CoA carboxylase (ACC1 and ACC2). ACC1 is cytosolic and regulates *de novo* fatty acid synthesis, while ACC2 is mitochondrial and is involved in fatty acid oxidation. *De novo* fatty acid synthesis has been shown to be increased in Dendritic cells (DCs) and macrophages in response to *M bovis* BCG, but in this model ACC1 or ACC2 inhibition in murine DCs and macrophages was not required to control infection (19). In CRISPR/Cas9 knock down experiments performed on ACC1 and ACC2 in BLaER1 cells (a human B and macrophage-like cell line), inhibition lowered foam cell formation and TAG levels in infected macrophages, while enhancing mitochondrial activity and limiting Mtb-induced necrotic cell death of macrophages (20). Mtb growth in ACC2, but not in ACC1 deficient cells was reduced compared to wild-type cells.

Of interest, Brandenburg et al. recently described an association between ACC2 and the Wnt family member 6 (WNT6). Immunohistochemical staining of human TB infected lung showed colocalized expression of WNT6 which correlated with Oil Red O signal. A similar colocalization was observed in the infected lungs of IL-13- overexpressing mice. WNT6- supported ACC2 activity increased intracellular TAG levels and Mtb survival in macrophages. A combination of ACC2 inhibitors with isoniazid improved the clinical outcome and reduced Mtb dissemination in mouse models of infection (20).

Although Foam cells in TB are predominantly TG rich, there are other components which are essential for any cellular membranes or droplet. Cholesterol esters are another component of droplets, generated by acyl-CoA: cholesterol *O*-acyltransferase (ACAT1 and ACAT2). The role of both enzymes is to process free cholesterol, derived from the diet or from endocytosed complex particles. ACAT1 and ACAT2 deficiency causes ER stress due to excess cholesterol (21, 22). ACAT1 is ubiquitous and abundant in macrophages, while ACAT2 is mainly expressed in hepatocytes and enterocytes in liver and intestine in the steady state (23). Both enzymes ACAT1 and ACAT2 are regulated by monocyte maturation into macrophages (24). ACAT2 positive macrophages appear in skin TB and sarcoidosis, among other pathologies (24). Recently, Genoula et al. found increased expression of CD36 and ACAT1 in human monocyte derived foam cells developed after exposure to TB-Pleural Effusions. This effect was IL-10 dependent (25). IL-10 activated STAT3 in these cells, which also bore high bacillary loads and showed an immunosuppressive phenotype as demonstrated by decreased production of TNF-α (25). We postulate that the inflammatory potential of foam cells might be a spectrum regulated by differences in TG and cholesterol accumulation.

## Foam Cells in TB Inflammation

Mtb interacts with a variety of macrophage receptors. Mycolic acids do not seem to form the bulk of the lipids of the foam cell, but they may play a role in foam cell formation and function by regulating lipid-related genes downstream of TLR and scavenger receptor pathways. For example, TLR2, CD14 and MARCO are required for murine and human macrophage cytokine responses to mycobacterial trehalose dimycolate and Mycobacterium tuberculosis (2). Mtb mannose-lipoarabinomannan (Man-LAM), lipomannan (LM), and mannosylated glycoproteins also interact with C-type lectin receptors (CLRs) such as Dectin-1, Mannose receptor, and DC-SIGN. Intracellular Nod-like receptors (NLRs) also participate in Macrophage recognition within the innate response to Mtb, reviewed in (26). Engagement of all these receptors can induce activation of intracellular kinases and of NF-kB and other inflammatory transcription factors, as well as lipid related PPAR receptors, leading to cell death and inflammation or survival and persistence, involving inflammatory cytokines and lipid related proteins.

An interesting autocrine loop mediated by TLR2 controls lipid droplet formation and TNF-α secretion by BCG-infected mouse peritoneal macrophages (27, 28). TLR2 stimulation by BCG triggers expression and activation of PPARγ and NF-kB. Inhibition of PPARγ by GW9662 inhibits droplet biogenesis, while inhibition of NF-kB by JSH-23 does not have any effect on lipid droplet accumulation (28). PPARγ- dependent lipid droplet accumulation depends on cooperation between TLR2 and CD36 with disruption resulting in the reduction of lipid droplets (28). Competition between the bacterium and the lipid ligands for CD36 and MARCO may bring about special signalling in foam cells, hitherto undefined.

Cytokines such as TNF-α act in an auto- and paracrine manner to promote droplet formation in Mtb infected human macrophages. TNFα recognition at the cell surface activates mTORC1 (mTOR complex 1) and caspase 8, which enhance lipid droplet formation by inhibition of lipophagy, promotion of mitochondrial dysfunction, and by activation of SREBP involvement in TAG synthesis (29). Furthermore, IL-10 in TB-PE promotes foamy phenotype in macrophages by activating STAT 3 (signal transducer and activator of transcription 3) which in turn upregulates the expression of ACAT, leading to cholesterol accumulation in lipid droplets (25). Additionally, foam cells also show enhanced expression of CD36, required for the import of extracellular lipid.

*In vitro*, using human monocyte derived macrophages, we found that foam cell formation prevented cells from dying. This may be due to activation of inflammasome versus death pathways in foam cells. In THP1 cells, which are more resistant to infection than primary macrophages, foam cells displayed an increased production of inflammatory cytokines including TNF-α, IL-1β, and an increased IL-1β/IL-10 ratio, compared with macrophages. Increased TNF-α secretion in response to Mtb infection of oleic acid-derived-THP-1-foam cells, involved the NF-κB pathway (30).

Foam cells can function as factories of inflammatory eicosanoids, producing arachidonic acid (AA)- derived eicosanoids such as prostaglandins, leukotrienes, thromboxanes, lipoxins, and related oxygenated lipid species, pro- or anti-inflammatory in nature. They can also produce resolvins and the balance between pro- and anti-inflammatory lipid mediators influences the outcome of infection. Lipid droplets are sites for many of the AA enzymes including cyclooxygenases and lipoxygenases. Specific GPCRs (G protein coupled receptors) sense these inflammatory lipids which act in a paracrine or autocrine manner. Examples include receptors for prostaglandin E2 and D2, leukotriene B4 and lipoxin (31). Fatty acids and leukotriene B4 bind directly to PPARα and PGJ2. Some derivatives of HETE bind PPARγ and modulate their effect on lipid metabolism (32). During Mtb infection, it has been demonstrated that pharmacological inhibition by both mepenzolate bromide (MPN) or siRNA, mediated reduction of GPR109A in THP-1 macrophages, resulting in less droplet formation and reduced bacterial loads (33). Infection of macrophages with Mtb or activation by ESAT-6, a virulence factor of Mtb, induces ketone body formation. Acetyl Co-A can be metabolised into 3HB (D-3-hydroxybutyrate) which activates GPR109A in a paracrine or autocrine manner to promote lipid droplet accumulation by exerting an antilipolytic effect (33). Fatty acids, intermediates of TAG synthesis such as diacylglycerol (DAG) and monoacylglycerol (MAG), acyl-coenzyme A and phosphatidic acid, are all potential signalling molecules in immunity (34).

## Final Considerations and Conclusions

The designation “foam cells” covers a range of storage, genetic, metabolic, inflammatory and infectious conditions. In this review we briefly covered the lipid content and metabolism associated with macrophage foam cells in TB. The roles of lipids in pathogenesis of florid, subclinical and latent disease are still poorly defined, nor do we have insights into their contribution to the hallmarks of granuloma formation such as Langhans giant cell formation, macrophage inflammasome and antimicrobial activation, epithelioid cell transformation and the induction of life threatening systemic acute and chronic inflammatory syndromes.

This review sets the stage for further studies of lipid metabolism in TB by temporal and spatial analysis of single cell gene expression in situ, exemplified by the recent publication by Russell and colleagues where both susceptibility of the macrophage to infection and the metabolic state of the bacterium were used as important variables in a single cell RNAseq study (35). Combined with the resurgence of interest in immunometabolism, the availability of human and experimental material will transform our understanding of the molecular and cellular mechanisms at play, the basis for improved therapy. To date the most convincing data emerge from studies that address human pathology, thus more efforts at obtaining samples should be made worldwide.

It remains to establish whether foam cell formation is a beneficial host response to Mtb infection, or another of the mechanisms Mtb uses to survive inside the cells by controlling their metabolic environment. Numerous studies suggest that Mtb can access host TAG and sequester it in the form of intracytoplasmic lipid inclusions (ILI) during persistence, as a source of carbon and energy (36, 37). Moreover, Mtb can also metabolize host membrane cholesterol for energy production and to maintain microbial cell wall components during persistence in IFN-γ activated macrophages, as in chronic animal models of infection (38).

A red flag arises when we see that for host lipid acquisition, Mtb upregulates many genes involved in lipid metabolism including Tgs-1, Ppe4- a perilipin like protein- involved in TAG synthesis, and Mce1, Mce4, LucA, OmamB, Mce1D, MceG, and *rv0966c*-, contributing to lipid import (36, 38–42). Mtb itself also expresses many genes involved in lipid hydrolysis such as lipY, Msh1, lipF, lipH, lipJ, lipK, lipN, lipV, lipX, lipY, culp5, culp7, and culp6, which may contribute to the breakdown of host lipid (40, 43, 44). Studies indicate that apart from using lipid as energy source, Mtb also utilises lipid for the synthesis and maintenance of its cell wall, largely consisting of lipids including mycolic acid, phiocerol-dimycoseroic acid, poly-acylated trehaloses and sulfolipids (37, 45).

The reason why only some macrophages undergo foam cell formation in the same environment where other macrophages differentiate towards epithelioid and giant cells, is unknown. To date there is no information about the developmental origin of foam cells in Mtb and the fact that foam cells form in many locations in the body would suggest a general tissue macrophage property or a feature of recruited blood monocytes in particular. In the lungs of TB patients, foam cells could originate from alveolar, interstitial, or recruited monocyte-derived macrophages, or specific subsets of these. The cell- specific phenotype, not necessarily their origin, was implicated by Ordway et al. who showed high expression of DEC-205 and TRAF1,2, 3, (TNFR-associated factors) in foam cells, the latter associated with resistance to apoptosis (46). Further research into the origin of foam cells in the lung and lymph nodes will be important.

There are reports which demonstrate that infected foam cells can leave the alveoli and reach the upper bronchus by the propelling movement of mucus, from where they can be swallowed or expectorated (47), thus facilitating Mtb dissemination and spread. During regression of atherosclerosis, foam cells may acquire characteristics of DC, shown by upregulated expression CCR7 allowing them to migrate to lymph nodes (48). In post-primary TB showing extensive lipid pneumonia, infected foam cells are prominent in exudates from disrupted granulomata, as well as in the lung alveoli, and Mtb organisms are found in close association with lipid droplets (5, 49).

Proteomic analysis of granulomata is also highly informative. Colocalization of LTA4H with TNFα predominantly in the marginal area of caseum suggests a strong association of these factors in inflammation and tissue necrosis (50). LTB4 and enzymes ALOX5, ALOX5AP, and LTA4H were more pronounced in the caseum, as well as in the cells adjacent to the caseum. Their expression diminished as the distance from the necrotic centre increased. They further observed that prostanoids and COX1 and COX2 were prominently located in the surrounding cells and less prevalent in the necrotic core. Establishing co-expression of lipid handling molecules, with lipid droplet positive macrophages, and the balance of pro and anti-inflammatory lipids in the foam cell reaction to Mtb, will be important. Development of proteomics and lipidomics applied to TB, will help this subject to progress. The main challenge for now is developing robust human and humanised models that enable investigators to extract and work with these labile cells, developing phenotypic markers for their characterization in combination with infection readouts to establish bona fide Foam cell functions.

## Author Contributions

PA, SG, and FM wrote the manuscript. PA wrote the first draft of the manuscript. FM revised the manuscript and edited. SG edited the manuscript. All authors contributed to the article and approved the submitted version.

## Conflict of Interest

The authors declare that the research was conducted in the absence of any commercial or financial relationships that could be construed as a potential conflict of interest.

## Publisher’s Note

All claims expressed in this article are solely those of the authors and do not necessarily represent those of their affiliated organizations, or those of the publisher, the editors and the reviewers. Any product that may be evaluated in this article, or claim that may be made by its manufacturer, is not guaranteed or endorsed by the publisher.

## Abbreviations

3HB, D-3-hydroxybutyrate; AA, Arachidonic acid; ACAT1,2, Acetyl-CoA acetyltransferase 1 and 2; ACC1, 2, Acetyl-CoA carboxylase 1 and 2; ACLY, ATP-citrate synthase; ACS, FattyAcylCo-A synthetase; ACSL1, Acyl-CoA Synthetase Long Chain Family Member 1; ADFP or ADRP, Adipose Differentiation-Related Protein, aliases for Perilipin 2 PLIN2; AFB, Acid-fast bacilli; AGPAT, 1-acyl glycerol-3-phosphate O-acyl transferase; ALOX5, Arachidonate 5-Lipoxygenase; ALOX5AP, Arachidonate 5-Lipoxygenase Activating Protein; BCG, Bacille Calmette-Guerin; BLaER1, B-cell-derived human cancer cell line; BMDM, Bone marrow derived macrophages; Cas9, CRISPR-associated protein 9; CCTα, Phosphate Cytidylyltransferase 1A, Choline, PCYT1A; CD14, CD14 molecule; CD163, CD163 molecule; CD206, CD206 molecule, Mannose receptor; CD36, CD36 molecule; CIC, Citrate carrier SLC25A1 Gene; cKO, Conditional Knockout; COX1, Cyclooxygenase 1, PTGS1 prostaglandin-endoperoxide synthase 1; COX2, Cyclooxygenase 2, PTGS2prostaglandin-endoperoxide synthase 1; cPLA2, Cytosolic Phospholipase A2, PLA2G4A Gene Phospholipase A2 Group IVA; CRISPR, Clustered Regularly Interspaced Short Palindromic Repeats; CS, Citrate synthase; culp5, Probable carboxylesterase Culp5; culp6, Carboxylesterase/lipase Culp6; culp7, Probable carboxylesterase Culp7; DAG, Diacylglycerol.; DCs, Dendritic cells; DEC-205, LY75 Gene; DGAT1,2, Diacylglycerol O-Acyltransferase 1 and 2; ER, Endoplasmic reticulum; FA, Fatty acid; FABP3,4,5 7, Fatty acid binding protein 3, 4, 5 or 7; FASN, Fatty acid synthase gene; FATP1, Fatty acid transport protein 1; GPAT, Glycerol-3-phosphate acyl transferase; GPCRs, G Protein coupled receptors; GPR109A, Hydroxycarboxylic Acid Receptor 1 HCAR1; GTP, Guanosine triphosphate; GW9662, Antagonist of PPARgamma; HCV, Hepatitis C virus; HETE, Hydroxyeicosatetraenoic acids; HIF-1a, Hypoxia Inducible Factor 1 Subunit Alpha; Hig-2, Hypoxia Inducible Lipid Droplet; HLADR, Human Leukocyte Antigen – DR, isotype; IFNγ, Interferon Gamma; IM54, Necrosis inhibitor; ILI, Intracytoplasmic lipid inclusions; JSH-23, Inhibitor of NF-κB transcriptional activity; KO, Knockout; LAL, Lysosomal acid lipases; lipF, Carboxylesterase LipF; lipH, Lipase LipH; lipJ, Probable lignin peroxidase LipJ; lipK, Hydrolase LipK; lipN, Carboxylic ester hydrolase LipN; lipV, Lipase LipV; lipX, Lipase LipX; lipY, Lipase LipY; LOX-1oxidized, Low-density lipoprotein receptor1; LPL, Lipoprotein lipase; LPS, Lipopolysaccharide; LTA4H, Leukotriene A4 Hydrolase; LTB4, Leukotriene B4; LucA, Rv3723/LucA integrates cholesterol and fatty acid uptake; MAPK, Mitogen-activated protein kinase; Marco, Macrophage receptor with collagenous structure; Mce1, Mce protein 1; Mce1D, Mce-family protein Mce1D; Mce4, Lipoprotein LprN; MceG, Putative ATPase subunit Rv0655/MceG; MPN, Mepenzolate bromide; MAG, Monoacylglycerol; Msh1, Mycobacterial secreted hydrolase 1; Mtb, Mycobacterium tuberculosis; Mtb, ESAT-6; mTORC1, mTOR complex 1; Myd88, Myeloid differentiation primary response 88; NF-Kb, Nuclear Factor kappa-light-chain-enhancer of activated B cells; OmamB, Putative components of the Mce1 fatty acid transporter (Rv0200/OmamB); oxLDL, Oxidized LDL; PAP, Phosphatidic acid phosphatase; PDL1, Programmed death-ligand 1; PGJ2, Prostaglandin J2; PLIN2, 3 Perilipin 2 and 3; PPARα, PPARγ, Peroxisome proliferator-activated receptor alpha or gamma; Ppe4, perilipin like protein 4; rv0966c, Uncharacterized protein Rv0966c; SAPC, SaposinC; Septin9, Septin-9, MLL septin-like fusion; siRNA, Silencing Ribonucleic Acid; SR-A1, Scavenger receptor class A type 1; SR-B1, Scavenger receptor class B type I; SREBP-1, Sterolbregulatory element binding protein 1; TAG, Triacylglycerol; TB, Tuberculosis; TDM, Lipid trehalose 6,6′-dimycolate of Mtb; Tgs-1, Probable diacyglycerol O-acyltransferase tgs1; THP1, Monocytic cell line; TLR2, Toll like receptor 2; TLRs, Toll like receptors; TNFα, Tumor Necrosis factor Alpha; TRAF1,2,3, TNFR associated factors; WHO, World Health Organization; WNT6, Wnt family member 6

## Glossary

**Table:**

3HB	D-3-hydroxybutyrate
AA	Arachidonic acid
ACAT1,2	Acetyl-CoA acetyltransferase 1 and 2
ACC1, 2	Acetyl-CoA carboxylase 1 and 2
ACLY	ATP-citrate synthase
ACS	FattyAcylCo-A synthetase
ACSL1	Acyl-CoA Synthetase Long Chain Family Member 1
ADFP or ADRP	Adipose Differentiation-Related Protein, aliases for Perilipin 2 PLIN2
AFB	Acid-fast bacilli
AGPAT	1-acyl glycerol-3-phosphate O-acyl transferase
ALOX5	Arachidonate 5-Lipoxygenase
ALOX5AP	Arachidonate 5-Lipoxygenase Activating Protein
BCG	Bacille Calmette-Guerin
BLaER1	B-cell-derived human cancer cell line
BMDM	Bone marrow derived macrophages
Cas9	CRISPR-associated protein 9
CCTα	Phosphate Cytidylyltransferase 1A
Choline	PCYT1A
CD14	CD14 molecule
CD163	CD163 molecule
CD206	CD206, molecule, Mannose receptor
CD36	CD36 molecule
CIC	Citrate carrier SLC25A1 Gene
cKO	Conditional Knockout
COX1	Cyclooxygenase 1
PTGS1	prostaglandin-endoperoxide synthase 1
COX2	Cyclooxygenase 2
PTGS2	prostaglandin-endoperoxide synthase 1
cPLA2	Cytosolic Phospholipase A2
PLA2G4A	Gene Phospholipase A2 Group IVA
CRISPR	Clustered Regularly Interspaced Short Palindromic Repeats
CS	Citrate synthase
culp5	Probable carboxylesterase Culp5
culp6	Carboxylesterase/lipase Culp6
culp7	Probable carboxylesterase Culp7
DAG	Diacylglycerol.
DCs	Dendritic cells
DEC-205	LY75 Gene
DGAT1,2	Diacylglycerol O-Acyltransferase 1 and 2
ER	Endoplasmic reticulum
FA	Fatty acid
FABP3,4,5 7	Fatty acid binding protein, 4, 5 or 7
FASN	Fatty acid synthase gene
FATP1	Fatty acid transport protein 1
GPAT	Glycerol-3-phosphate acyl transferase
GPCRs	G Protein coupled receptors
GPR109A	Hydroxycarboxylic Acid Receptor 1 HCAR1
GTP	Guanosine triphosphate
GW9662	Antagonist of PPARgamma
HCV	Hepatitis C virus
HETE	Hydroxyeicosatetraenoic acids
HIF-1a	Hypoxia Inducible Factor 1 Subunit Alpha
Hig-2	Hypoxia Inducible Lipid Droplet
HLADR	Human Leukocyte Antigen – DR isotype
IFN-γ	Interferon Gamma
IM54	Necrosis inhibitor
ILI	Intracytoplasmic lipid inclusions
JSH-23	Inhibitor of NF-κB transcriptional activity
KO	Knockout
LAL	Lysosomal acid lipases
lipF	Carboxylesterase LipF
lipH	Lipase LipH
lipJ	Probable lignin peroxidase LipJ
lipK	Hydrolase LipK
lipN	Carboxylic ester hydrolase LipN
lipV	Lipase LipV
lipX	Lipase LipX
lipY	Lipase LipY
LOX-1oxidized	Low-density lipoprotein receptor1
LPL	Lipoprotein lipase
LPS	Lipopolysaccharide
LTA4H	Leukotriene A4 Hydrolase
LTB4	Leukotriene B4
LucA	Rv3723/LucA integrates cholesterol and fatty acid uptake
MAPK	Mitogen-activated protein kinase
Marco	Macrophage receptor with collagenous structure
Mce1	Mce protein 1
Mce1D	Mce-family protein Mce1D
Mce4	Lipoprotein LprN
MceG	Putative ATPase subunit Rv0655/MceG
MPN	Mepenzolate bromide
MAG	Monoacylglycerol
Msh1	Mycobacterial secreted hydrolase 1
Mtb	Mycobacterium tuberculosis
Mtb	ESAT-6
mTORC1	mTOR complex 1
Myd88	Myeloid differentiation primary response 88
NF-Kb	Nuclear Factor kappa-light-chain-enhancer of activated B cells
OmamB	Putative components of the Mce1 fatty acid transporter (Rv0200/OmamB)
oxLDL	Oxidized LDL
PAP	Phosphatidic acid phosphatase
PDL1	Programmed death-ligand 1
PGJ2	Prostaglandin J2
PLIN2, 3	Perilipin 2 and 3
PPARα, PPARγ	Peroxisome proliferator-activated receptor alpha or gamma
Ppe4	perilipin like protein 4
rv0966c	Uncharacterized protein Rv0966c
SAPC	SaposinC
Septin9	Septin-9
MLL	septin-like fusion
siRNA	Silencing Ribonucleic Acid
SR-A1	Scavenger receptor class A type 1
SR-B1	Scavenger receptor class B type I
SREBP-1	Sterolbregulatory element binding protein 1
TAG	Triacylglycerol
TB	Tuberculosis
TDM	Lipid trehalose 6,6′-dimycolate of Mtb
Tgs-1	Probable diacyglycerol O-acyltransferase tgs1
THP1	Monocytic cell line
TLR2	Toll like receptor 2
TLRs	Toll like receptors
TNF-α	Tumor Necrosis factor Alpha
TRAF1,2,3	TNFR associated factors
WHO	World Health Organization
WNT6	Wnt family member 6
